# Analysis of hospitalization expenses and influencing factors for elderly cancer patients in a tertiary hospital in Dalian, China: a five‑year retrospective study

**DOI:** 10.1186/s12885-024-12635-6

**Published:** 2024-07-18

**Authors:** Lilin Zhang, Xijing Zhuang, Xiumei Yang, Feng Xu, Nan Wang, Zhanfang Guo, Junfeng Chen, Ding Ding

**Affiliations:** 1 Medical Department, Central Hospital of Dalian University of Technology, Dalian, 116033 China; 2 Group Work Department, Central Hospital of Dalian University of Technology, Dalian, 116033 China; 3 College of Public Health, Dalian Medical University, Dalian, 116044 China

**Keywords:** Elderly, Cancer, Hospitalization expenses, Influencing factors

## Abstract

**Background:**

Because the proportion of elderly individuals and the incidence of cancer worldwide are continually increasing, medical costs for elderly inpatients with cancer are being significantly increasing, which puts tremendous financial pressure on their families and society. The current study described the actual direct medical costs of elderly inpatients with cancer and analyzed the influencing factors for the costs to provide advice on the prevention and control of the high medical costs of elderly patients with cancer.

**Method:**

A retrospective descriptive analysis was performed on the hospitalization expense data of 11,399 elderly inpatients with cancer at a tier-3 hospital in Dalian between June 2016 and June 2020. The differences between different groups were analyzed using univariate analysis, and the influencing factors of hospitalization expenses were explored by multiple linear regression analysis.

**Results:**

The hospitalization cost of elderly cancer patients showed a decreasing trend from 2016 to 2020. Specifically, the top 3 hospitalization costs were material costs, drug costs and surgery costs, which accounted for greater than 10% of all cancers according to the classification: colorectal (23.96%), lung (21.74%), breast (12.34%) and stomach cancer (12.07%). Multiple linear regression analysis indicated that cancer type, surgery, year and length of stay (LOS) had a common impact on the four types of hospitalization costs (*P* < 0.05).

**Conclusion:**

There were significant differences in the four types of hospitalization costs for elderly cancer patients according to the LOS, surgery, year and type of cancer. The study results suggest that the health administration department should enhance the supervision of hospital costs and elderly cancer patient treatment. Measures should be taken by relying on the hospital information system to strengthen the cost management of cancer diseases and departments, optimize the internal management system, shorten elderly cancer patients LOS, and reasonably control the costs of disease diagnosis, treatment and department operation to effectively reduce the economic burden of elderly cancer patients.

Cancer is one of the most common chronic diseases, and its annual incidence and mortality are rapidly increasing worldwide [1, 2]. According to the global report on cancer by the World Health Organization (WHO) in 2020, there were approximately 19.3 million new cancer cases and 10 million related deaths. It is estimated that the number of new cancer cases worldwide will reach 28.4 million in 20 years, and the number of global cancer deaths will increase 59%, from 7.4 million in 2004 to 11.8 million deaths by 2030 [3, 4]. Cancer has become the second leading cause of death globally [5]. Cancer is commonly observed in older adults, and the incidence of cancer in elderly individuals is 8.47 times higher than young individuals [6]. Approximately 64% of cancer patients globally will be aged ≥ 60 years [7]. Recent studies have projected a worldwide doubling of new cancers diagnosed in adults aged > 65 years and a tripling in adults aged > 80 years in the coming decades [8, 9].

Cancer causes high medical costs for patients. For some families, the cost may be catastrophic. The global annual cost of cancer treatment is estimated to be US$1.16 trillion [10]. However, cancer costs for the European Union were estimated to be €51 billion, 40% of which were direct health care costs [11]. The direct medical cost of cancer care in the United States reached US$77.4 billion and increased to US$124.57 billion a decade ago [12, 13]. In England, colorectal, breast, prostate and lung cancer costs are estimated to be US$2.34 billion annually for hospital care alone [14]. However, the annual medical cost of cancer treatment in China is over US$32.4 billion [15]. The growth rate of medical expenses per capita in the elderly population is greater and increased faster than other age groups [16]. With increasing age, the health status and medical needs of the elderly also change [17], and the impact of population aging on the medical expenses of elderly cancer patients has become a major challenge for the global health care system [18, 19].

According to the latest global cancer burden data released by the International Agency for Research on Cancer (IARC) of the WHO, nearly 19.29 million new cancer cases occur worldwide, and 4.57 million cases (23.7%) originate in China. The number of cancer-related deaths reached 9.96 million, and 3 million of these cases (30%) originated in China [20]. China has the highest cancer incidence worldwide, and it has become the country that is most affected by cancer [21, 22]. The elderly are the main patient group affected by cancer, and the high incidence and mortality rates of cancer in these patients and the large medical expenses incurred over time have a profound impact on the Chinese economy [23–25]. It places a heavy burden on families and the social care of elderly cancer patients [26].

Dalian is a subprovincial city in Liaoning Province located at the southern end of the Liaodong Peninsula, with a geographical area of 12,574 km^2^. The per capita gross domestic product was 105,046 Chinese Yuan (CNY) (approximately 6907 dollars) in 2021, and the economic development was at a medium level according to measurements in China. It has abundant medical resources, and the city had 3516 health institutions (excluding village clinics) and 49,068 beds in 2020 [27]. One of the resulting sociodemographic changes in Dalian is the dramatic increase in the number of older adults (aged ≥ 60 years). According to the seventh national census, the resident population of Dalian was 7.54 million, and the proportion aged 60 years and over was 24.71% [28]. The proportion of the aging population in Dalian ranks third among the 35 major cities in China. Therefore, we chose Dalian as a representative research city.

In summary, actively addressing the economic burden of cancer in elderly people has become an urgent task worldwide. Most domestic studies on cancer hospitalization costs are limited to a single disease type, but a few studies have analyzed the changes and structural composition of cancer hospitalization costs from the perspective of the elderly population and multiple disease types. Therefore, the present study used the retrospective data of elderly patients with cancer aged 60 years and older from 2016 to 2020 in a tertiary hospital in Dalian to describe the actual direct medical costs of inpatients with cancer and its components according to the inventories of patients’ hospitalization costs and analyze the influencing factors for the costs to provide advice on the reduction and control of the high medical costs of elderly patients with cancer.

## Material and methods

### Data resources and samples

The data used in this study were extracted from a hospital-based information system, which is an important part of the hospital system. We used a retrospective observational design to investigate 11,399 elderly patients who were diagnosed with cancer between June 2016 and June 2020 (International Classification of Diseases 10th revision: C20). Eligible inpatients were those who met the following inclusion criteria: (1) were 60 years or older; (2) had a primary diagnosis of cancer; and (3) had complete basic information, clinical features and related cost information. The following exclusion criteria were used: (1) patients with a hospital stay shorter than 1 day or longer than 100 days; (2) patients for whom the code could not be identified; and (3) elderly inpatients for whom key information was missing. According to the inclusion and exclusion criteria, the sample sizes from 2016 to 2020 were 1875, 2020, 2209, 2249 and 1985, respectively, and the final sample size was 10,338.

### Study variables

Cancer includes a series of diseases that are classified by the International Classification of Diseases (ICD). Since some specific diseases could not be classified, 10 disease categories were classified after the exclusion of these data. Therefore, the composition ratio of cancer type was 100% and included colorectal cancer, lung cancer, breast cancer, cancer of the stomach and other cancer types. The collected data included sociodemographic characteristics, clinical characteristics and different types of costs incurred during the hospitalization period [29, 30]. Sociodemographic characteristics included sex, age, the year of admission, and type of medical insurance, which included Urban Employee Basic Medical Insurance (UEBMI), Urban Resident Basic Medical Insurance (URBMI), and other types of insurance [31–33]. Clinical characteristics consisted of the patient’s access to the hospital, the LOS, whether the patient underwent surgery and the disease type [34–36]. Total hospitalization costs consisted of costs for medicine (Western medicine and Chinese traditional medicine), surgeries, medical consumables (low-value and high-value supplies), diagnoses (pathological diagnosis, laboratory diagnosis, CT scans, digital imaging and magnetic resonance imaging diagnosis, clinical diagnosis), and other costs (e.g., blood infusions) [37, 38].

### Statistical analysis

The entire statistical analysis process may be divided into 4 steps. First, descriptive analysis was used to provide a detailed description of the demographic and clinical disease characteristics of elderly cancer patients, and continuous variables are described as the means ± standard deviation (SD). Second, to avoid the influence of time on the results, we used all of the expense data reported in CNY based on the 2020 values, which were inflated using the year-specific personal health care consumer price index (CPI) of China. Third, because the cost data were typically positively skewed, Mann-Whitney u test (for 2 groups) and the Kruskal-Wallis h test (for more than 2 groups) were used to account for non-normally distributed data and examine significant differences between groups [39]. Finally, multiple linear regression analysis was performed on log-transformed medical expenses to determine possible risk factors [40, 41].

The different hospitalization costs of elderly inpatients with cancer were the dependent variable (y), and the possible influencing factors were the independent variables (x). However, the magnitude of the influence of each independent variable (x) on the dependent variable (y) in the same model was determined by the normalized coefficient of the variable |β|. The current study selected 8 possible influencing factors: (1) sex; (2) age; (3) type of medical insurance; (4) year of admission; (5) access to hospital; (6) the LOS; (7) surgery status; and (8) type of cancer. Statistical analyses were performed using SPSS Statistics for Windows, version 26.0. For all comparisons, differences were tested using two-tailed tests, and a p value less than 0.05 was deemed statistically significant.

## Results

### Basic information of elderly inpatients with cancer

A total of 10,338 elderly patients were included. More than half of the patients were male (54.65%), and the sex ratio was 1.20:1. The largest proportion of patients were aged 60–69 years, which accounted for 55.19%. The largest subset of patients were urban employees (74.72%). Most patients were admitted through outpatient clinics (84.74%). The LOS for most patients was 10–30 days (60.81%). Cancer type was dominated by colorectal, lung, breast and stomach cancers, with a cumulative percentage of 70.11% (Table 1).

**Table 1:** Demographic characteristics of the elderly cancer patients in 2016–2020

Variable	Groups	Number of cases (*n* = 10,338)	Proportion (%)
Gender	Male	5650	54.65
	Female	4688	45.35
Age	60–69	5706	55.19
	70–79	3062	29.62
	≥ 80	1570	15.19
Insurance	UEBMI	7725	74.72
	URBMI	1995	19.30
	Others	618	5.98
Access to hospital	Emergency	1578	15.26
	Outpatient	8760	84.74
The LOS (days)	< 10 days	3746	36.24
	10–30 days	6287	60.81
	> 30 days	305	2.95
Surgery	No	1991	19.26
	Yes	8347	80.74
Type of cancer	Colorectal cancer	2477	23.96
	Lung cancer	2247	21.74
	Breast cancer	1276	12.34
	Stomach cancer	1248	12.07
	Bladder cancer	745	7.21
	Kidney cancer	581	5.62
	Prostate cancer	544	5.26
	Thyroid cancer	474	4.58
	Liver cancer	470	4.55
	Ovarian cancer	276	2.67

### Analysis of the composition ratio of hospitalization costs of elderly patients with cancer in different years

From 2016 to 2020, the average hospitalization costs of all elderly patients with cancer in tertiary hospitals showed a trend of first decreasing then increasing, and the highest hospitalization costs occurred in 2016 (RMB 38114.10). Compared to 2016, the average hospitalization cost decreased by 3.83% in 2020. This study divided hospitalization costs into 5 parts: drug costs, surgery costs, material costs, diagnosis costs and other costs. During 2016–2020, the average hospitalization costs were as follows: materials, RMB 13269.18 (37.15%); drugs, RMB 8247.98 (23.09%); surgery, RMB 5575.96 (15.61%); diagnosis, RMB 5425.95 (15.19%); and other costs, RMB 3202.85 (8.96%).

For the hospitalization costs, the proportions of surgery costs and other costs gradually increased, and the proportion of drug costs gradually decreased. The changes in the proportions of material costs and diagnosis costs first decreased then increased (Table 2).

**Table 2:** Distribution and composition ratio of hospitalization costs of elderly patients with cancer in 2016–2020

Variable	2016 (yuan)	2017 (yuan)	Trend	2018 (yuan)	Trend	2019 (yuan)	Trend	2020 (yuan)	Trend	Total (yuan)
Drug costs	11,467.49 ± 9195.49	8730.74 ± 7473.82	↓***	7032.47 ± 6335.23	↓***	7273.92 ± 6295.13	↑*	7171.91 ± 6414.75	↓*	8247.98 ± 7348.36
Material costs	13,916.26 ± 14,794.54	13,762.04 ± 14,200.80	↓	12,284.77 ± 12,833.92	↓**	13,063.33 ± 12,812.30	↑**	13,485.15 ± 13,284.27	↑	13,269.18 ± 13,570.90
Diagnosis costs	5453.86 ± 2803.37	4985.55 ± 2761.31	↓***	5121.03 ± 2888.76	↑	5550.36 ± 3111.38	↑***	6046.15 ± 2874.39	↑***	5425.95 ± 2919.36
Surgery costs	4440.15 ± 4021.94	4983.02 ± 4409.90	↑**	5702.87 ± 4982.74	↑***	6145.40 ± 4967.83	↑**	6465.79 ± 5354.69	↑***	5575.96 ± 4840.78
Others	2836.34 ± 3064.37	2931.93 ± 3192.94	↑	3338.25 ± 4409.57	↑***	3369.98 ± 3239.60	↑***	3484.71 ± 3728.52	↑	3202.85 ± 3586.29
Total	38,114.10 ± 28,487.36	35,393.27 ± 26,834.58	↓ **	33,479.39 ± 25,651.27	↓*	35,402.99 ± 25,539.82	↑***	36,653.72 ± 26,374.04	↑	35,721.93 ± 26,570.63

Z test result values: Drug costs: 2016vs2017(-9.059); 2017vs2018(-7.768); 2018vs2019(-2.520); 2019vs2020(-1.578); Material costs: 2016vs2017(-0.058); 2017vs2018(-3.110); 2018vs2019(-2.672); 2019vs2020(-1.275); Diagnosis costs: 2016vs2017(-5.409); 2017vs2018(-1.603); 2018vs2019(-4.534); 2019vs2020(-5.634); Surgery costs: 2016vs2017(-2.899); 2017vs2018(-5.127); 2018vs2019(-2.609); 2019vs2020(-3.646); Others costs: 2016vs2017(-1.394); 2017vs2018(4.446); 2018vs2019(-3.738); 2019vs2020(-1.210); Total costs: 2016vs2017(-3.178); 2017vs2018(-2.228); 2018vs2019(-3.485); 2019vs2020(-1.213)

↑ increasing trend; ↓ decreasing trend; Reference group *p < 0.05, **p < 0.01, ***p < 0.001 compared between 2016 and 2017, 2017 and 2018, 2018 and 2019, 2019 and 2020 (two-tailed test)

### Hospitalization costs for different types of elderly cancer patients

#### Basic information on disease composition, hospitalization costs and LOS

For all cancer system diseases, the average hospitalization cost was 35,721.93 yuan. The average hospitalization cost for stomach cancer patients was the highest (RMB 58514.71), and the cost for breast cancer patients was the lowest (RMB 16138.69). Other cancers with average hospitalization costs greater than RMB 30,000 included colorectal cancer (RMB 49435.32), lung cancer (RMB 41367.76), kidney cancer (RMB 34798.62), and ovarian cancer (RMB 31117.96). For different cancer disease inpatient days, the average LOS for all cancers was 13.22 days. Stomach cancer patients had the greatest average hospitalization duration (16.39 days), and breast cancer patients had the least average hospitalization duration (7.32 days). Excluding thyroid and breast cancer, the average length of hospital stay for patients with other cancers was longer than 10 days. Among all elderly cancer diseases, the proportion of thyroid cancer patients receiving surgery was the highest at 98.52%, and the proportion of liver cancer patients receiving surgery was the lowest at 57.66%. More than 80% of cancer patients who underwent surgery had breast cancer (96.24%), bladder cancer (94.90%), ovarian cancer (90.58%), kidney cancer (87.95%), colorectal cancer (84.74%), and prostate cancer (82.54%) (Table 3).

**Table 3:** Basic information on diseases of elderly patients with cancer in 2016–2020

Type	Hospitalization costs (yuan)	Rank	The LOS (days)	Surgery proportion (%)
Colorectal cancer	49,435.32 ± 29,037.23	2	15.39 ± 9.39	84.74
Lung cancer	41,367.76 ± 25,223.18	3	13.09 ± 7.73	68.94
Breast cancer	16,138.69 ± 5968.96	10	7.32 ± 4.35	96.24
Stomach cancer	58,514.71 ± 30,089.29	1	16.39 ± 10.51	71.15
Bladder cancer	27,426.07 ± 16,031.17	7	14.55 ± 7.81	94.90
Kidney cancer	34,798.62 ± 12,889.96	4	13.36 ± 4.59	87.95
Prostate cancer	28,202.59 ± 20,207.92	6	14.47 ± 7.20	82.54
Thyroid cancer	20,990.87 ± 6002.13	9	8.07 ± 3.82	98.52
Liver cancer	24,419.66 ± 21,798.81	8	11.88 ± 8.49	57.66
Ovarian cancer	31,117.96 ± 16,216.23	5	12.53 ± 5.81	90.58

#### Average hospitalization costs and composition of diseases that account for more than 10% of all cancers

**Fig. 1:**
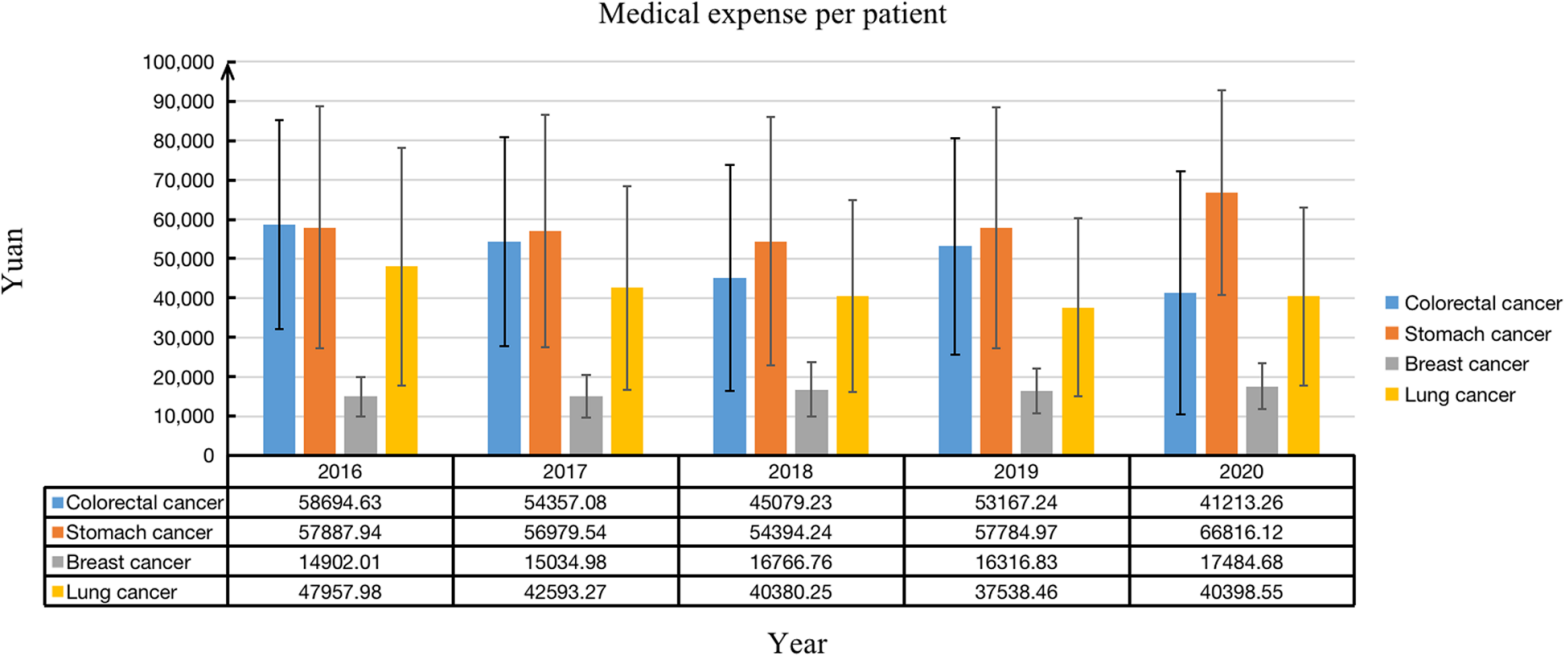
Trends in hospitalization costs for top four types of elderly cancer patients in 2016–2020

From 2016–2020, diseases that account for more than 10% of all cancer diseases included colorectal (23.96%), lung (21.74%), breast (12.34%) and stomach (12.07%) cancers. Among the average hospitalization costs for these four types of cancer, stomach cancer had the highest average hospitalization cost (RMB 58514.71). In contrast, breast cancer patients had the lowest average hospitalization costs (RMB 16138.69). According to the overall trend of the average hospitalization costs for the four cancers, colorectal cancer and lung cancer showed a downward trend, with an average annual decrease of 8.46% and 4.20%, respectively. However, the average hospitalization costs for breast cancer and stomach cancer patients showed a gradual upward trend, with an average annual growth of 3.65% and 4.08%, respectively (Fig. 1).

**Fig. 2:**
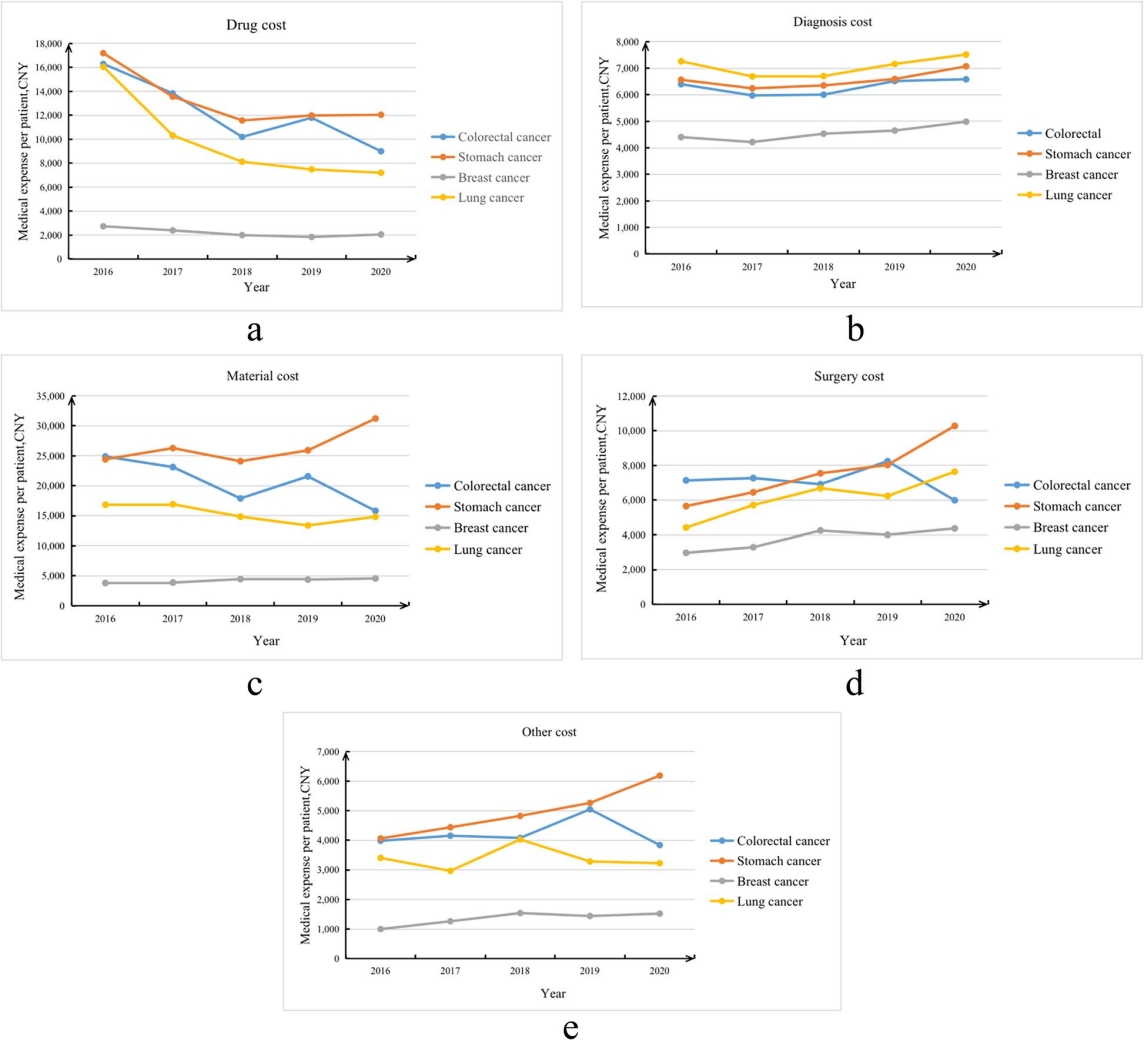
**a**-**e** The distribution of different hospitalization costs of elderly cancer patients in 2016–2020 (**a** Trends in drug costs; **b** Trends in diagnosis costs; **c** Trends in material costs; **d** Trends in surgery costs; **e** Trends in other costs)

For the hospitalization costs for the four types of cancers, the proportion of drug costs has been decreasing annually, and the proportions of surgery, material, diagnosis and other costs have generally increased (Fig. 2a-e).

For drug costs, the average hospitalization cost for stomach cancer patients was the highest (RMB 13294.38), the average hospitalization cost for breast cancer patients was the lowest (RMB 2180.16), and the average annual reduction rates were as follows: lung cancer (18.16%), colorectal cancer (13.79%), stomach cancer (8.50%) and breast cancer (6.86%) (Fig. 2a).

For diagnosis costs, the average hospitalization cost for patients with lung cancer was the highest (RMB 7073.60), and the average hospitalization cost for patients with breast cancer was the lowest (RMB 4557.98). The average annual growth rates were 3.14% for breast cancer, 1.88% for stomach cancer, 0.86% for lung cancer, and 0.71% for colorectal cancer (Fig. 2b).

For material costs, the average hospitalization cost for stomach cancer patients was the highest (RMB 26252.14), and the average hospitalization cost for breast cancer patients was the lowest (RMB 4220.67). There was an upward trend in the cost of stomach cancer and breast cancer with average annual growth rates of stomach cancer (6.34%) and breast cancer (4.69%). However, there was a downward trend in the cost of colorectal cancer and lung cancer with average annual decrease rates of 10.73% for colorectal cancer and 3.13% for lung cancer (Fig. 2c).

For surgery costs, the average hospitalization cost for stomach cancer patients was the highest (RMB 7504.85), and the average hospitalization cost for breast cancer patients was the lowest (RMB 3811.05). In addition to the downward trend in the cost of colorectal cancer, the other three types of cancer surgery costs showed an upward trend. The average annual growth rates were as follows: stomach cancer (16.13%), lung cancer (14.69%), and breast cancer (10.10%) (Fig. 2d).

For other costs, the average hospitalization cost for stomach cancer patients was the highest (RMB 4917.75), and the average hospitalization cost for breast cancer patients was the lowest (RMB 1368.83). There was an upward trend in the cost of stomach cancer and breast cancer, and the average annual growth rates were 11.10% for stomach cancer and 11.09% for breast cancer. However, there was a downward trend in the cost of lung cancer and colorectal cancer with an average annual decrease rate of 1.33% for lung cancer and 0.92% for colorectal cancer (Fig. 2e).

### Analysis of hospitalization costs in elderly cancer patients with different disease characteristics that account for more than 10% of all cancers

Gender, age, insurance type, hospital access, LOS (days) and surgery status were used as independent variables. These expenses were considered the dependent variable. Among all of the expense categories, year and cancer type were significantly associated with medical expenses (*P* < 0.05). For total cost and material cost, univariate analysis revealed that gender, age, access to hospital, LOS (days) and surgery status was performed were significantly associated with medical expenses (*P* < 0.05). For drug cost, univariate analysis showed that gender, age, insurance type, access to hospital, LOS (days) and surgery status was performed were significantly associated with medical expenses (*P* < 0.05). For diagnosis cost, univariate analysis showed that gender, age, insurance type, LOS (days) and surgery status was performed were significantly associated with medical expenses (*P* < 0.05) (Table 4).

**Table 4:** One-way analysis of factors affecting hospitalization costs of elderly cancer patients

Variable	Groups	Total cost (yuan)	Drug cost (yuan)	Material cost (yuan)	Diagnosis cost (yuan)
Gender	Male	48,212.11 ± 29,449.01	11,659.82 ± 8497.17	19,318.88 ± 15,395.22	6611.39 ± 2908.99
	Female	36,481.31 ± 26,932.98	7502.18 ± 7307.62	14,062.55 ± 13,659.46	5881.02 ± 2659.80
	Z	15.413***	23.555**	10.645***	11.653***
Age	60~69	39,997.79 ± 27,392.77	8705.29 ± 7660.32	15,975.32 ± 14,535.70	6085.25 ± 2625.23
	70~79	45,523.51 ± 29,539.71	10,519.76 ± 8539.94	18,017.41 ± 15,007.74	6541.63 ± 2898.68
	≥ 80	46,500.76 ± 32,298.98	11,664.60 ± 9140.29	17,254.30 ± 15,302.88	6353.16 ± 3330.49
	H	52.191***	114.177***	14.385**	33.484***
Insurance type	UEBMI	42,902.37 ± 29,381.85	9847.19 ± 8442.33	16,827.34 ± 15,033.12	6327.54 ± 2875.66
	URBMI	39,935.08 ± 27,326.57	8294.54 ± 7175.70	15,497.41 ± 14,017.05	6259.32 ± 2612.37
	Others	41,818.34 ± 27,090.03	9338.76 ± 7555.33	17,042.93 ± 14,147.61	5896.77 ± 2604.37
	H	5.788	15.463***	4.708	27.652***
Access to hospital	Emergency	55,334.33 ± 27,899.40	13,201.58 ± 8344.37	22,691.99 ± 14,585.86	6311.90 ± 3284.63
	Outpatient	40,087.00 ± 28,384.86	8959.07 ± 8003.97	15,645.07 ± 14,580.51	6240.84 ± 2718.10
	Z	14.364***	17.231***	11.781***	0.217
The LOS (days)	< 10	16,608.27 ± 12,170.40	3003.95 ± 2576.12	4880.25 ± 6555.35	4731.78 ± 2065.19
	10~30	53,724.56 ± 24,062.24	12,154.08 ± 6817.28	22,395.74 ± 13,999.96	6859.19 ± 2618.46
	> 30	89,847.11 ± 26,212.53	27,170.09 ± 11,411.86	30,894.19 ± 13,675.82	9931.71 ± 4092.33
	H	3200.859***	3341.919***	2240.622***	1247.057***
Surgery	Yes	46,126.51 ± 28,521.65	10,077.91 ± 8250.96	18,750.11 ± 14,635.39	6454.75 ± 2736.27
	No	15,326.15 ± 11,422.10	6195.53 ± 6929.45	1896.39 ± 2180.86	4760.69 ± 2917.28
	Z	30.404***	14.704***	36.054***	14.484***

Reference group **p < 0.01, ***p < 0.001 (two-tailed test)

### Risk factors for the four types of hospitalization costs in elderly cancer patients for diseases that account for greater than 10% of all cancers

Because the four types of hospitalization costs were skewed, the log-transformed of these costs was considered the dependent variable to ensure the normal distribution of the costs. Multivariate analysis was performed using factors that were significant in the univariate analysis. The results showed that in the total cost stepwise regression model, gender, LOS, surgery status, access to hospital, year, colorectal cancer, breast cancer, and stomach cancer were significant determinants of hospitalization costs (*P* < 0.05). According to the standardized coefficients, the factors associated with total costs, in decreasing order, were LOS, surgery status, breast cancer, access to hospital, stomach cancer, year, gender, and colorectal cancer (adjusted *R*^2^ = 0.645) (Table 5). For the drug costs, stepwise regression model, age, access to hospital, surgery status, year, LOS, UEBMI, breast cancer, and stomach cancer were significant determinants of hospitalization costs (*P* < 0.05). According to standardized coefficients, the factors associated with drug costs decreased in the following order: LOS, breast cancer, surgery status, year, access to hospital, stomach cancer, UEBMI and age (adjusted *R*^2^ = 0.579) (Table 6). For the material costs stepwise regression model, gender, age, access to hospital, surgery status, year, LOS, colorectal cancer, breast cancer, stomach cancer were significant determinants of hospitalization costs (*P* < 0.05). According to the standardized coefficients, the factors associated with material costs, in decreasing order, were surgery, LOS, breast cancer, stomach cancer, year, access to hospital, colorectal cancer, gender, and age (adjusted *R*^2^ = 0.539) (Table 7). For the diagnosis costs stepwise regression model, surgery status, year, LOS, breast cancer, stomach cancer, and colorectal cancer were significant determinants of hospitalization costs (*P* < 0.05). According to the standardized coefficients, the factors associated with diagnosis costs decreased in the following order: LOS, surgery, breast cancer, colorectal cancer, stomach cancer, and year (adjusted *R*^2^ = 0.533) (Table 8).

**Table 5:** Multiple regression model of total hospitalization costs

Variable	B	SE	β	T	*P*	95%CI
Colorectal cancer^a^	-0.025	0.007	-0.032	-3.524	0.000	-0.038 to -0.011
Breast cancer	-0.285	0.009	-0.301	-30.774	0.000	-0.303 to -0.267
Stomach cancer	0.071	0.009	0.072	8.247	0.000	0.054 to 0.088
Gender	0.025	0.006	0.034	4.007	0.000	0.013 to 0.037
Age	0.000	0.000	-0.001	-0.162	0.871	-0.001 to 0.001
Access to hospital	-0.080	0.008	-0.079	-10.188	0.000	-0.096 to -0.065
Surgery	0.457	0.009	0.403	51.442	0.000	0.440 to 0.474
Year	-0.012	0.002	-0.045	-5.970	0.000	-0.016 to 0.008
The LOS	0.021	0.000	0.501	60.353	0.000	0.020 to 0.021

*SE* Standard error, *β beta* Standardized coefficient, *T* T-statistic, *P P*-value, α = 0.05, *CI* Confidence intervals, a: The reference group is lung cancer

**Table 6:** Multiple regression model of drug hospitalization costs

Variable	B	SE	β	T	*P*	95%CI
URBMI^a^	0.016	0.018	0.009	0.919	0.358	-0.018 to 0.051
UEBMI	0.033	0.012	0.027	2.738	0.006	0.009 to 0.056
Colorectal cancer	-0.011	0.011	-0.010	-1.023	0.306	-0.032 to 0.010
Breast cancer	-0.554	0.014	-0.409	-38.415	0.000	-0.583 to -0.526
Stomach cancer	0.054	0.013	0.038	4.065	0.000	0.028 to 0.081
Gender	-0.008	0.010	-0.008	-0.825	0.410	-0.027 to 0.011
Age	0.001	0.001	0.020	2.399	0.016	0.000 to 0.003
Access to hospital	-0.111	0.012	-0.076	-8.943	0.000	-0.135 to -0.086
Surgery	0.295	0.014	0.182	21.367	0.000	0.268 to 0.322
Year	-0.049	0.003	-0.129	-15.359	0.000	-0.055 to -0.043
The LOS	0.026	0.001	0.436	48.191	0.000	0.025 to 0.027

^a^The reference group is others

**Table 7:** Multiple regression model of material hospitalization costs

Variable	B	SE	β	T	*P*	95%CI
Colorectal cancer	0.073	0.013	0.056	5.420	0.000	0.046 to 0.099
Breast cancer	-0.318	0.018	-0.199	-17.917	0.000	-0.352 to -0.283
Stomach cancer	0.228	0.016	0.137	13.834	0.000	0.195 to 0.260
Gender	0.062	0.012	0.050	5.159	0.000	0.038 to 0.085
Age	-0.002	0.001	-0.027	-3.141	0.002	-0.004 to -0.001
Access to hospital	-0.112	0.015	-0.065	-7.418	0.000	-0.142 to -0.082
Surgery	0.945	0.017	0.497	55.649	0.000	0.912 to 0.979
Year	-0.031	0.004	-0.069	-7.947	0.000	-0.038 to -0.023
The LOS	0.025	0.001	0.363	38.384	0.000	0.024 to 0.026

**Table 8:** Multiple regression model of diagnosis hospitalization costs

Variable	B	SE	β	T	*P*	95%CI
URBMI	0.013	0.011	0.014	1.098	0.272	-0.010 to 0.035
UEBMI	0.006	0.008	0.011	0.842	0.400	-0.009 to 0.021
Colorectal cancer	-0.092	0.007	-0.169	-13.166	0.000	-0.105 to -0.078
Breast cancer	-0.150	0.009	-0.223	-15.988	0.000	-0.168 to -0.131
Stomach cancer	-0.075	0.009	-0.108	-8.813	0.000	-0.092 to -0.059
Gender	0.006	0.006	0.011	0.931	0.352	-0.007 to 0.018
Age	-0.001	0.000	-0.015	-1.415	0.157	-0.001 to 0.000
Surgery	0.239	0.009	0.299	26.714	0.000	0.222 to 0.257
Year	0.010	0.002	0.053	4.848	0.000	0.006 to 0.014
The LOS	0.010	0.000	0.349	29.449	0.000	0.009 to 0.011

## Discussion

Cancer has become a disease that seriously threatens the health of elderly people in developed and developing countries [42]. With the increase in the aging population, the expected total cost of cancer will increase significantly [43, 44]. An increasing number of elderly patients have come to hospitals for cancer treatment because the high incidence of cancer has led to an increase in the number of patients. Therefore, the cost of cancer treatment has become a direct financial burden for elderly patients and their families [45]. The present study analyzed the distribution characteristics and influencing factors of in-hospital charges for various types of cancer in elderly individuals.

Our study showed that colorectal cancer, lung cancer, breast cancer and stomach cancer accounted for more than 10% of all cancer diseases in the classification, which differs slightly from the latest global cancer burden data on major cancer composition and ranked by the IARC of the WHO [20]. The top two cancers are the same, and the third and fourth most common cancers were stomach cancer and liver cancer nationwide. The ranks of breast and bladder cancers in this study were greater than the national level, and the ranks of liver and ovarian cancers were relatively low. For hospitalization costs, the average hospital expenses of all elderly cancer patients showed a decreasing trend annually. The significant increase in medical expenses of hospitalized elderly cancer patients was reasonably controlled during 2016–2020. Multiple hospital measures are needed to reduce the medical costs of elderly cancer patients, and the problem of “difficult and expensive medical treatment” for the elderly population has been controlled, which effectively reduce the financial burden of elderly cancer patients and their families. Our study revealed that the average surgery ratio was 86.43%, and the average LOS for elderly cancer patients was 14.08 days, which indicates that most elderly cancer patients with more serious conditions require surgery, are more difficult to treat and have longer treatment courses. Factors such as postoperative complications, postoperative rehabilitation and the condition of elderly cancer inpatients may be closely related to hospitalization duration.

From 2016–2020, material costs, drug costs and surgical costs constituted a relatively large proportion of inpatient costs for elderly cancer patients, with material costs accounting for the largest proportion at 37.15%. This result is similar to a study in Zhejiang province in China [46]. One possible reason is the recent rapid increase in the prices of medical consumables, particularly those that are imported. Additionally, there has been a noticeable trend among elderly cancer patients to choose more expensive consumables during surgical treatments. Furthermore, as most medical consumables are currently priced mainly by enterprises on their own, the lack of effective regulatory and review, which also resulted in inflated prices. For total costs, the proportion of drug costs decreased significantly, from a maximum of 29.45% in 2016 to 19.19% in 2020. However, the current hospitalization cost for drugs in malignant inpatients remains significantly lower than the global average of 16% [47]. Previous studies conducted in Serbia and other developing country in Southeast Europe has explored similar cost ratio [48, 49]. This result may be attributed to the Liaoning Provincial Government issuing the “Implementation Opinions on Continuously Promoting the Deepening of the Reform of the Medical and Health System” in 2017, which required all urban public hospitals in the province to impose zero markup on drug costs and encouraged the use of inexpensive medications. With the abolishment of pharmaceutical markups, the proportion of drug costs in relation to total hospitalization costs will be reduced. Therefore, the policy driver orientation is highly significant. In contrast, expenditures for diagnoses and surgery, which reflect the value of medical labor, were relatively low in the overall medical expenditure. The diagnostic costs increased annually from 13.30% in 2016 to 16.38% in 2020. The A.M. AGUS research revealed that the proportion of diagnostic costs in the composition of first-year hospital costs for cancer patients in Northern Ireland was similar to the present study [50]. One possible reason is that the treatment of elderly cancer patients requires a perfect examination, such as screening, diagnosis, and staging, all of which rely on the assistance of medical imaging technology. The number of items to examine is increasing, especially for advanced-stage elderly patients with cancer recurrence, metastasis and complications. As elderly patients’ awareness of self-care and health insurance reimbursement increases, they continue to pursue more comprehensive and accurate examinations in the course of treatment, and excessive examinations increase costs. Surgery costs increased annually from a minimum share of 11.13% in 2016 to 16.66% in 2020, which showed a similar trend with the study of hospital costs for cancer patients in Italy [51]. The reason may be the improvements in cancer treatment methods (such as immunotherapy and targeted therapy), other new technologies and the introduction of advanced instruments and equipment in recent years.

The multiple linear regression model indicated that cancer type, surgery status, year and LOS had a common impact on the four types of hospitalization costs (*P* < 0.05). First, the study revealed that hospitalization duration was the most influential factor on hospitalization costs. As the length of hospital stay increased for elderly cancer patients, the cost of hospitalization increased. This finding aligns closely with Erica Manrriquez’s research on the factors influencing hospitalization expenses for elderly cancer patients in the United States [52]. The average hospitalization cost of elderly cancer patients discharged after 10~30 days was 3–4 times greater than patients discharged after < 10 days. The following reasons may explain this observation. Longer hospitalization days, more serious conditions or long-term treatment needs require more resources to be consumed, which leads to higher hospitalization costs. However, excessive unnecessary preoperative tests and inefficient hospital clinical pathway management may also lead to longer hospitalization stays and higher hospitalization costs for elderly cancer patients [53].

Second, elderly cancer patients who underwent surgery had significantly greater hospital costs than non-surgery patients. Chen et al reached a similar conclusion [54]. This relationship is primarily due to the use of high-value medical materials. For elderly cancer patients with the same disease, the cost of materials is much greater for elderly patients who choose surgery than for non-surgery elderly patients. The proportion of material costs for surgical elderly patients with lung cancer was 38.75%, and the proportion of material costs used in the non-surgical setting was only 12.33%. Additionally, elderly cancer patients undergoing surgery always have complex treatment processes that require more medical consumables and more expensive materials.

Third, there was a significant difference in the cost of hospitalization for elderly cancer patients between different years, with a decreasing trend from 2016 to 2018 but an increasing trend in 2019 and 2020. The reason why costs have been somewhat controlled in previous years may be due to the promulgation of the document “Notice on the Issuance of Several Opinions on Controlling Unreasonable Growth of Medical Costs in Public Hospitals” issued by the National Health and Wellness Commission, which makes controlling the unreasonable growth of medical costs in public hospitals an important goal and task in deepening medical reform, reasonably adjusting the costs of medical services, reducing the costs of drugs and materials, and optimizing the revenue and expenditure structure of public hospitals to achieve benign hospital operation. Moreover, the hospitalization expenses in 2019 and 2020 fluctuated, which may be related to the COVID-19 outbreak. During this period, elderly people, as a vulnerable group, were prone to exacerbation of the condition of some elderly cancer patients due to a variety of factors, such as physical decline and weakened immune function [55, 56], which led to increased hospitalization costs. According to the requirements of epidemic prevention and control work, under the conditions of limited hospital beds and hospitalization days, we must make every effort to ensure timely treatment of critically ill elderly patients. Therefore, the number of critically ill elderly cancer patients seeking medical treatment and hospitalization in hospitals has increased compared to previous years, which has led to an increase in hospitalization costs.

Finally, the results of the present study showed that different types of cancer have significant effects on hospitalization costs. For example, hospitalization costs for stomach cancer patients were significantly greater than for breast cancer patients. The main cost components of the two types of cancer were surgery and chemotherapy, but the resource use profiles differed. One possible reason may be that different types of cancer require distinct treatment methods, and the cost of stomach cancer treatment is much greater than breast cancer treatment. Elderly patients with stomach cancer have twice as many hospitalization days as patients with breast cancer, which resulted in significantly greater hospitalization costs for elderly patients with stomach cancer.

There were also some unique influencing factors. For example, gender differences in the total cost of hospitalization were found, and the total hospitalization costs were greater in males than females. Previous research by Jaqueline Avila has explored similar hospital expenses for elderly cancer patients [57]. The following reasons may explain the gender difference in hospitalization costs. First, the incidence and severity of disease are different because the disease spectra vary between sexes. Compared to women, men are more likely to have unhealthy lifestyles, such as smoking and alcohol abuse [58, 59], which also aggravated the occurrence and development of disease burdens in males. Second, men are under greater social and family stress that will exacerbate the risk of illness. However, the study revealed that the drug costs of older cancer patients (≥ 80 years old) were greater than relatively younger elderly cancer patients (60–79 years old). The reason may be related to the poor resistance of older cancer elderly patients (≥ 80 years old) and slower postoperative recovery, which result in a longer duration of disease and more difficult treatment [60]. Therefore, older cancer patients (≥ 80 years old) need more drugs for treatment. For material costs, there was a significant difference between elderly cancer patients with different access to hospitals, and the material costs for emergency patients were higher than outpatients. This finding differed from Ian Flemingin’s study on hospitalization costs for cancer patients in Northern Ireland [61]. This difference is primarily due to emergency hospitalization of elderly inpatients, who were likely to have more serious conditions and a relatively high percentage of surgeries, which increases material costs compared to outpatients.

## Strengths and limitations

The strengths of this study include the use of recent 5 years of continuous hospital data to analyze the hospitalization costs of different types of elderly cancer patients and the large sample size. Inflation between years was also considered. This study provides information that may be used in tailored therapeutic interventions and economic evaluations for elderly patients with cancer.

There were some limitations in this study. First, we only collected direct medical cost information for elderly cancer patients, which did not include information on indirect and intangible costs. Therefore, we are likely underestimating the total medical costs of elderly cancer patients. Second, some clinical information, such as cancer severity, comorbidities, and pathological type, were omitted from the analysis because these data were not available at the time. This lack of additional data leads to limitations in the multiple regression analysis. Finally, the research was based on a single hospital, which may not reflect the cost of hospitalization for elderly patients with cancer all over the country.

## Conclusions

The results of this study revealed a declining trend in hospitalization costs for elderly cancer patients from 2016 to 2020. However, there are common significant differences in the four types of hospitalization costs for elderly cancer patients according to the LOS, surgery, year and type of cancer. Based on this study, we can conclude that the health administration department should increase its investment in the supervision of hospital costs and elderly cancer patients treatment. Measures should be taken to rely on the hospital information system to strengthen the cost management of cancer patients and departments, optimize the internal management system, shorten the LOS of elderly cancer patients, and reasonably control the costs of disease diagnosis, treatment and department operation to effectively reduce the economic burden on elderly cancer patients.

## Data Availability

The datasets used and/or analyzed during the current study are available from the corresponding author on reasonable request.

## Abbreviations

LOS: Length Of Stay
CNY: Chinese Yuan
ICD: International Classification of Diseases
SD: Standard Deviation
CPI: Consumer Price Index
COVID-19: Corona Virus Disease 2019
WHO: World Health Organization
UEBMI: Urban Employee Basic Medical Insurance
URBMI: Urban Resident Basic Medical Insurance
IARC: International Agency for Research on Cancer
